# Bromide of Potassium in Sickness of Pregnancy

**Published:** 1869-04

**Authors:** 


Bromide of Potassium in Sickness of Pregnancy.—By Dr. Packard. (*Amer. Jour. Med Sciences*, July.)

Dr. Packard states that he has successfully employed the bromide in several cases where the sickness has been manifestly due to reflex irritation of the stomach, and in which the other remedies had failed. He gives twenty grains every three hours until the symptoms begin to yield.

